# An Overview of Immunotherapeutic Approaches Against Canine Visceral Leishmaniasis: What Has Been Tested on Dogs and a New Perspective on Improving Treatment Efficacy

**DOI:** 10.3389/fcimb.2019.00427

**Published:** 2019-12-18

**Authors:** Ana Alice Maia Gonçalves, Jaqueline Costa Leite, Lucilene Aparecida Resende, Reysla Maria da Silveira Mariano, Patricia Silveira, Otoni Alves de Oliveira Melo-Júnior, Helen Silva Ribeiro, Diana Souza de Oliveira, Diogo Fonseca Soares, Thaiza Aline Pereira Santos, Alexandre Ferreira Marques, Alexsandro Sobreira Galdino, Olindo Assis Martins-Filho, Walderez Ornelas Dutra, Denise da Silveira-Lemos, Rodolfo Cordeiro Giunchetti

**Affiliations:** ^1^Laboratory of Biology of Cell Interactions, Department of Morphology, Institute of Biological Sciences, Federal University of Minas Gerais, Belo Horizonte, Brazil; ^2^Laboratory of Immuno-Proteome and Parasite Biology, Department of Parasitology, Institute of Biological Sciences, Federal University of Minas Gerais, Belo Horizonte, Brazil; ^3^Laboratory of Biotechnology of Microorganisms, Federal University of São João Del-Rei, Divinópolis, Brazil; ^4^Laboratory of Diagnostic and Monitoring Biomarkers, René Rachou Institute, FIOCRUZ-Minas, Belo Horizonte, Brazil

**Keywords:** canine visceral leishmaniasis, *Leishmania infantum*, biomarkers, treatment, immunotherapy

## Abstract

Visceral leishmaniasis (VL), caused by digenetic protozoa of the genus *Leishmania*, is the most severe form of leishmaniasis. *Leishmania infantum* is one of the species responsible for VL and the disease caused is considered a zoonosis whose main reservoir is the dog. Canine visceral leishmaniasis (CVL) can lead to the death of the animal if left untreated. Furthermore, the available pharmocologial treatment for CVL presents numerous disadvantages, such as relapses, toxicity, drug resistance, and the fact treated animals continue to be reservoirs when treatment fails to achieve parasitological cure. Moreover, the available VL control methods have not been adequate when it comes to controlling parasite transmission. Advances in immune response knowledge in recent years have led to a better understanding of VL pathogenesis, allowing new treatments to be developed based on immune system activation, often referred to as immunotherapy. In fact, well-defined protocols have been described, ranging from the use of immunomodulators to the use of vaccines. This treatment, which can also be associated with chemotherapy, has been shown to be effective in restoring or inducing an adequate immune response to reduce parasitic burden, leading to clinical improvement. This review focuses on immunotherapy directed at dogs infected by *L. infantum*, including a literature review of what has already been done in dogs. We also introduce a promising strategy to improve the efficacy of immunotherapy.

## Introduction

Leishmaniasis is a group of infectious parasitic diseases caused by protozoa of the *Leishmania* genus (Rossi and Fasel, 2017). Visceral leishmaniasis (VL) is the most severe form, which can result in a high mortality rate in humans if untreated (Alemayehu and Alemayehu, 2017). It is known that three species are responsible for causing VL; *Leishmania (Leishmania) donovani* (Laveran and Mesnil, 1903) and *Leishmania (Leishmania) infantum* (Nicolle, 1908) are found in the Old World, while *Leishmania* (*Leishmania*) *chagasi* (Cunha and Chagas, 1937) is found in the New World. Although they have different names and different geographical origins, molecular data suggest that *L. infantum* and *L. chagasi* are the same species (Maurício et al., 2000).

In recent years, cases of human VL have been reported in 76 countries (Organização Pan-Americana da Saúde, 2018) and, in 2017, 95% of the new cases occurred in seven countries: Brazil, Ethiopia, India, Kenya, Somalia, South Sudan, and Sudan (World Health Organization, 2018). Brazil accounts for 96% of the number of human VL cases in Latin America (Organização Pan-Americana da Saúde, 2018).

The VL, caused by *L. infantum*, is a zoonosis in which the dog (*Canis familiaris*) serves as the main domestic reservoir (World Health Organization, 2010; Roatt et al., 2014; Duarte et al., 2016). The disease in dogs may be manifested by inducing apparent clinical signs that, when present, may range from mild to severe, causing death (Maia-Elkhoury et al., 2008; Reis et al., 2009). During VL urbanization (Da Silva et al., 2017), dogs became responsible for spreading the disease throughout the Brazilian countryside, resulting in a rising number of human VL cases (Reis et al., 2010). Notably, cases of canine visceral leishmaniasis (CVL) precede human cases (Leite et al., 2018).

The applied VL control measures are not adequate when it comes to interrupting the spread of the disease. Moreover, *Leishmania* antigens are not able to induce a high immunogenicity regarding protection against infection in dogs (Giunchetti et al., 2019). Although, CVL treatment cannot induce parasite clearance, this measure has been largely employed, thus demonstrating the dogs' close relationship in our society. In this sense, immunotherapeutic treatments have shown to be promising against CVL, with the main objective of reestablishing dog immunity and, therefore, parasite control (Roatt et al., 2017). This approach can be performed alone or in combination with chemotherapy (Singh and Sundar, 2014). The focus of this review is on the immunotherapy methods already described for the CVL treatment, whether or not associated with chemotherapy. Taking into account the complexity of CVL transmission, we discuss some current aspects regarding immunology, resistance and susceptibility biomarkers, as well as available control measures and disease treatment.

## General Aspects of the Immunological Profile and Biomarkers Regarding Susceptibility and Resistance in Canine Visceral Leishmaniasis

The immune response in CVL is of great importance for understanding the pathogenesis of the disease (Alvar et al., 2004; Ribeiro et al., 2018; Giunchetti et al., 2019). The immune response profile can trigger a resistance or susceptibility pattern during the parasite infection, resulting in different clinical forms of the disease (Moreno and Alvar, 2002; Leal et al., 2014; Giunchetti et al., 2019).

With regard to vector contact with the canine host, in addition to local lesion formation induced by vector feeding (Solano-Gallego et al., 2001; Giunchetti et al., 2006; Jacintho et al., 2018), the deposition of infective *L. infantum* promastigotes takes place in the dermis along with salivary content vector. This process recruits phagocytic cells to the site, such as neutrophils, macrophages, and dendritic cells, creating a pro-inflammatory environment (Soulat and Bogdan, 2017).

An *in vitro* study demonstrated that neutrophils are effector cells with the ability to control the initial infection, resulting in reduced parasite viability (Pereira et al., 2017). Furthermore, it has been observed that neutrophils have an ability to produce high levels of IFN-γ when stimulated with soluble antigen of *L. infantum* (Leal et al., 2014). Moreover, other molecules of the innate immunity have been correlated with ongoing CVL, such as TLRs (Toll-like receptors) (Hosein et al., 2015; Pereira-Fonseca et al., 2017) and chemokines (Menezes-Souza et al., 2012; Solcà et al., 2016).

It is known that the main immune response against the parasite is induced by the adaptive response, especially the type 1 immune response, characterized by IFN-γ, TNF-α, and IL-2 production related to the resistance profile. This type of immune response is related to the upregulation of the anti-leishmanial activity in macrophages (Koutinas and Koutinas, 2014), this being the main effector mechanism of the intracellular death of *Leishmania* amastigotes (Baneth et al., 2008). In this sense, the type 1 immune response induces cytokines, such as IFN-γ and TNF-α, predominant in asymptomatic dogs, demonstrating their protective potential against the disease (Costa-Pereira et al., 2015). Solano-Gallego et al. (2016) demonstrated that infected dogs presenting high levels of IFN-γ had lower parasite loads when compared to infected dogs that did not produce this cytokine. Dogs lacking this cytokine have more severe clinical symptoms, with higher parasitemia (Martínez-Orellana et al., 2017). Similarly, Th17 cells induce *L. infantum* control growth (Nascimento et al., 2015; Rodriguez-Cortes et al., 2017).

In contrast, the type 2 immune response, characterized IL-4, IL-5, IL-10, and TGF-β cytokines, is related to susceptibility in CVL (Sanches et al., 2014; Rodríguez-Cortés et al., 2016; Rodriguez-Cortes et al., 2017; Rossi et al., 2016; Solano-Gallego et al., 2016; Solcà et al., 2016; Tonin et al., 2016; De Martini et al., 2018). These susceptible dogs manifest a common pattern in the progression of clinical signs, with severity and variety of signs increasing with disease progression, in which most clinicopathological changes become evident after 12 months of infection (Foglia Manzillo et al., 2013). The type 2 immune response provides an anti-inflammatory cytokine microenviroment deactivating the cellular immune response against *L. infantum* infection (Rodriguez-Cortes et al., 2017). Moreover, a pronounced anti-*Leishmania* humoral response leads to the production of high levels of non-immunoprotective antibodies (Barbiéri, 2006; Gradoni, 2015), highlighting the polyclonal B cell response characteristic of susceptibility in CVL (Koutinas and Koutinas, 2014). There is still no consensus as to which IgG subclass is related to resistance or susceptibility in CVL (Lima et al., 2017; Chaabouni et al., 2018). Furthermore, excessive activation of humoral immunity may lead to the production of autoantibodies (Koutinas and Koutinas, 2014), such as antiactin and antitubulin (Pateraki et al., 1983), antinuclear (Smith et al., 2004; Ginel et al., 2008), and antitransferrin (Chaabouni et al., 2018).

**Figure 1:**
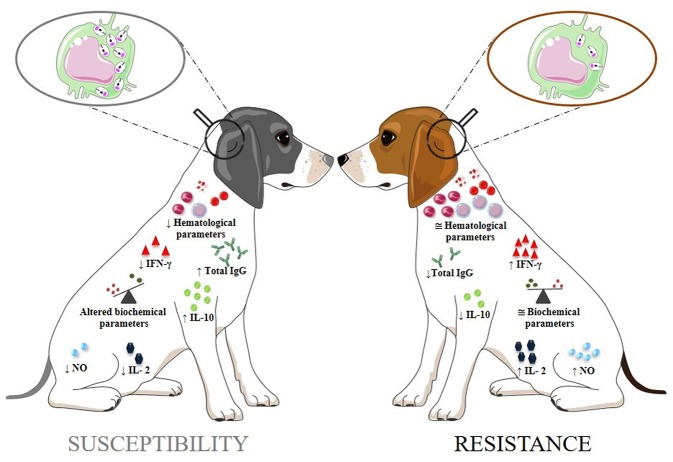
The biomarkers of canine visceral leishmaniasis related to susceptibility or resistance. The arrows (↑ and ↓) indicate the increase and decrease in biomarker levels, respectively; **≅**: approximate normal levels; **↓** Hematological parameters: decreased in red blood cells, lymphocytes, eosinophils, and platelets; Altered Biochemical parameters: hyperproteinemia, hypoalbuminemia, increased in aspartate aminotransferase, alanine aminotransferase, alkaline phosphatase, urea, and creatinine levels.

Although the cellular and humoral immunity parameters help to understand the progression of CVL, as well as the mechanisms related to resistance or susceptibility, integrated studies of several biomarkers are needed for a better understanding of the disease (Solcà et al., 2016). In asymptomatic dogs, hematological and biochemical parameters usually remain unchanged, while in symptomatic dogs changes may occur (Maia and Campino, 2018). Symptomatic dogs showed a significant decrease in red cells, lymphocytes, eosinophils, and platelets (Lopes et al., 2018). The biochemical parameters can be used to assess the general health status in CVL. Ongoing CVL is characterized by hyperproteinemia, hypoalbuminemia, and changes in aspartate aminotransferase, alanine aminotransferase, alkaline phosphatase, urea, and creatinine concentrations (Heidarpour et al., 2012; Ribeiro et al., 2018). These parameters are interesting markers for therapeutic monitoring, especially those related to the kidney, since damage to this organ associated with the disease is almost unavoidable (Ribeiro et al., 2018). All of the biomarkers included in this section and regarding resistence or susceptibility in CVL are summarized in Figure 1.

## Current Control Methods Based on Sandfly Interference to Block Canine Visceral Leishmaniasis Transmission

The approach to visceral leishmaniasis control needs to consider all elements in the transmission network, such as (i) sandfly vector, (ii) parasite reservoirs, and (iii) human health. In this sense, health control and surveillance measures, based on the Brazilian National Visceral Leishmaniasis Program of the Ministry of Health, determine: (i) the use of chemical insecticides and (ii) environmental management for vector population control and vector-human contact reduction, (iii) canine serological surveys, (iv) euthanasia of positive cases and timely diagnosis, and (v) adequate treatment of human cases to prevent severe forms of disease and death (Ministério da Saúde, 2014). However, it has been reported that an urgent revision in this control program is required, as its effectiveness ranges from low to moderate (Werneck et al., 2014).

In an attempt to reduce the adaptation of the vector population to the peridomic environment, the environmental management associated with chemical spraying can be used as a preventive action (Lara-Silva et al., 2017). However, this strategy is unsustainable in the long term due to the size of the area to be treated (Otranto and Dantas-Torres, 2013). The use of insecticides/repellents (mainly pyrethroids), impregnated in dog collars or used for individual human protection on the skin and/or clothing (Alexander and Maroli, 2003) aims to prevent contact with the vector. Deltamethrin, lead representative, impregnated in dog collars induced a reduction from 53 to 59% in the CVL incidence rate of infected sandflies (Kazimoto et al., 2018). In addition, uncollared dogs showed a higher frequency of clinical signs with faster progression when compared to collared dogs, demonstrating the anti-feeding effect (Foglia Manzillo et al., 2006), presenting an interesting combination of disease control and cost-effectiveness (Shimozako et al., 2017). Another type II pyrethroid, Flumethrin, applied pour-on in dogs resulted in a significant reduction on total mortality rate and in the blood-feeding index of sandflies (Jalilnavaz et al., 2016). Furthermore, the systemic insecticide Fluralaner (Bravecto®, MSD animal health) (Gomez and Picado, 2017; Miglianico et al., 2018) used in dogs has demonstrated induction of 40–60% mortality of phlebotomines using a membrane feeding assay (Gomez et al., 2018a) and 90% mortality when the vector was direct feeding (Gomez et al., 2018b). Moreover, sandfly feeding in vaccinated dogs with CaniLeish® resulted in a marked reduction in *Phlebotomus perniciosus* infection (Bongiorno et al., 2013).

Recently, a newly patented vaccine using non-salivary antigens from sandflies has shown promise as a vector control strategy because it impairs its life cycle in addition to blocking *Leishmania* infection in sandflies. This approach has been considered as the next vaccine frontier for controlling vector-borne diseases (Graciano et al., 2019).

Despite all existing control measures, preventing the spread of VL has been ineffective in Brazil (Romero and Boelaert, 2010). In this context, researchers advocate alternative control measures, such as mass vaccination and treatment of dogs, since these approaches are able to induce reduction in the parasite load and block *L. infantum* transmission in sandflies, thus providing evidence for reducing new canine and human VL cases (Pessoa-e-Silva et al., 2019).

## Conventional Canine Visceral Leishmaniaisis Treatment

Treatment of CVL is characterized by high rates of relapse, regardless of the antileishmanial drugs used, either as a single drug or in combined drug therapy (Ribeiro et al., 2018). Moreover, clinical and parasitological cure is rarely achieved, not to mention the possibility of drug resistance (Travi, 2014; Marcondes and Day, 2019).

Drug therapy using miltefosine was originally developed as an anticancer agent in the 1990s and was first recorded for VL treatment in 2002 in India (Dorlo et al., 2012). In 2016, the Brazilian Ministry of Health and the Ministry of Agriculture Livestock and Supply approved the registration of Milteforan® (Virbac, Brazil) (Brasil, 2016). Although there was a notable improvement in the clinical symptoms when using this drug, it was not accompanied by parasitological clearance, suggesting that treatment with miltefosine should not be recommended (Andrade et al., 2011). Recently, miltefosine treatment against CVL revealed clinical improvement with a reduction in infectivity from *L. infantum-*infected dogs (Dos Santos Nogueira et al., 2019).

Allopurinol has a parasitostatic activity and its long-term administration maintains low parasite loads, thus contributing to the prevention of canine relapse (Koutinas et al., 2001). The association of this drug with miltefosine showed to be a promising combination for CVL treatment (Foglia Manzillo et al., 2009). However, induced resistance is also a problem associated with the use of allopurinol (Yasur-Landau et al., 2017).

In most parts of the world, meglumine antimoniate is the most commonly used treatment for human and canine leishmaniasis. Meglumine antimonate, combined with allopurinol, is considered the most effective therapy for CVL (Solano-Gallego et al., 2009); however, CVL treatment with the same human-used drugs is not recommended since it may induce parasite resistance (Travi, 2014).

The great challenge of CVL treatment is to identity a drug that (i) is not used in VL human treatment, (ii) does not induce kidney damage or any other adverse effect, (iii) provides a parasite load control, (iv) interferes in the sandflies' life cycle, and (v) blocks parasite transmission. In this sense, other treatment options should be studied, such as immunotherapy, in an attempt to improve CVL treatment efficacy.

## Immunotherapy and Immunochemotherapy as Strategies for Improving Canine Visceral Leishmaniasis Treatment Efficacy

Immunotherapy involves the use of biological substances or molecules to modulate immune responses for the purpose of achieving prophylactic and/or therapeutic success (Okwor and Uzonna, 2009; Musa et al., 2010; Khadem and Uzonna, 2014; Roatt et al., 2014; Singh and Sundar, 2014). For instance, immunotherapeutic agents exert their effect by directly or indirectly augmenting the host's natural defenses, restoring the impaired effector functions or reducing the host's excessive response (Oldham and Smalley, 1983; Okwor and Uzonna, 2009).

Since *Leishmania* is able to persist in host cells by evading or exploiting their immune mechanisms, the ability to develop a specific immune response could induce parasite replication control (Gupta et al., 2013). Thus, triggering the immune system with antigens or immunomodulators could be an alternative approach to combatting distinct infections such as leishmaniasis (Scott and Novais, 2016). In fact, cutaneous leishmaniasis (CL) immunotherapy treatment was evaluated by Avila et al. (1982) using glucan immunotherapy, but without satisfactory results. In Brazil, the first study was carried out by Badaro et al. (1990), which demonstrated the immunotherapeutic ability of IFN-γ when concomitantly administered with pentavalent antimony in human visceral leishmaniasis. Notably, Mayrink et al. (1992) proposed immunotherapy using a mixture of five *Leishmania* strains and observed a 76% cure rate in human CL.

Distinct therapeutic approaches in CVL discussed in this section are summarized in Table 1. Since immunotherapeutic treatment against *Leishmania* infection has been successfully proved, the first study in dogs was performed by Neogy et al. (1994) using LiF2 antigen alone or combined with N-methylglucamine antimonate. These authors described that the immunochemotherapy protocol was more efficient for CVL treatment, demonstrating a 100% clinical cure rate, in which they did not observe any parasite in direct microscopic examination of bone-marrow aspirates. Another study demonstrated that the association of N-methyl D-glucamine antimoniate and *L. infantum* antigens (soluble antigen) showed an increase in the proportion of T lymphocytes; however, lymphnode aspirates remained positive (Guarga et al., 2002). Treatment using *L. braziliensis* promastigotes, alone or in association with Glucantime®, showed that chemotherapy alone was more effective, since the dogs had the lowest parasite load (Melo et al., 2002). Similarly, the *L. major* promastigote antigens and heat-killed *Mycobacterium vaccae* (SRL172) were compared to Glucantime® chemotherapy and revealed that both treatments were able to control parasitism, albeit slower in immunotherapy than in chemotherapy treatment (Jamshidi et al., 2011).

**Table 1:** Major immunotherapy and immunochemotherapy treatments evaluated in dogs against *L. infantum* infection.

**Country**	**Type of infection/number of animals in the study**	**Immunotherapeutic agent/treatment scheme/number of animals**	**Chemotherapeutic agent/treatment scheme**	**Treatment efficacy/improvements**	**References**
Corsica (French)	Naturally infected symptomatic dogs/24 animals	LiF2 antigen/3 IM doses at 7-day intervals/8 animals	Glucantime®/20 doses of 300 mg/kg by IM at 2-day intervals	100% cure rate	Neogy et al., 1994
Spain	Naturally infected dogs/10 animals	Soluble antigen of *L. infantum*/3 SC doses at 14- day intervals/5 animals	Glucantime®/21 consecutive doses of 100 mg/kg by SC	↑ proportion of T lymphocytes (CD4/TcRαβ^+^ and CD4/CD45RA^+^) in PBMCs	Guarga et al., 2002
Brazil	*L. infantum* experimentally infected with 1 × 10^7^ amastigotes. Treatment starts at 150 dpi/32 animals	Dead promastigote of *L. brasiliensis*/3 SC cyles of 20 days with 10-day intervals/8 animals	Glucantime®/3 cyles of 20 days of 100 mg/kg by SC with 10-day intervals	↓ efficacy when compared with the group treated only with chemotherapy	Melo et al., 2002
Brazil	Naturally infected asymptomatic dogs/67 animals	FML-vaccine/3 doses/21 animals	–	Positive DTH response in 79–95% of the animals. Absence of parasite in bone marrow smears	Borja-Cabrera et al., 2004
Italy	Naturally infected asymptomatic dogs/15 animals	Leish111f+ MPL®-SE/3 SC doses at 28- day intervals with second three-dose after 1 year/9 animals	–	7 out of 9 animals progressed to a subsequent stage of infection, detected by PCR of bone marrow, lymph node aspiration, and serology	Gradoni et al., 2005
Brazil	*L. infantum* experimentally infected with 2 × 10^8^ amastigotes. Treatment starts at 180 dpi/24 animals	enriched-Leishmune®/3 SC doses at 20- to 30-day intervals/12 animals	–	75% of the animals presented positive DTH with lower clinical scores and normal CD4+ counts	Santos et al., 2007
Brazil	Naturally infected symptomatic dogs/30 animals	Leish-110f® + MPL-SE/3 SC doses at 21-day intervals/6 animals	Glucantime®/2 cyles of 10 days of 100 mg/kg by IM with 10-day intervals	↓ deaths ↑ survival; specific cellular reactivity	Miret et al., 2008
Spain	Naturally infected dogs/98 animals	Domperidone/1 mg/Kg by OR every 12 h during 30 days/98 animals	–	Clinical improvement in 86% of animals with serum antibody titres decreased by 38%	Gómez-Ochoa et al., 2009
Brazil	Naturally infected symptomatic dog/59 animals	Leish-111f® + MPL-SE/4 SC doses at 7-day intervals/18 animals	Glucantime®/Daily doses of 20 mg/kg by IV during 30 days	75% cure rate in group treated only with immunotherapy	Trigo et al., 2010
Iran	*L. infantum* experimentally infected with 3 × 10^5^ amastigotes. Treatment starts at 60 dpi/19 animals	*Leishmania major* antigen+ heat-killed *Mycobacterium vaccae*/3 ID doses at 30-day intervals/3 animals	Glucantime®/ 30 consecutive doses of 100 mg/kg by IM	Complete clearance of parasite with no relapse in the group treated only with immunotherapy	Jamshidi et al., 2011
Brazil	Naturally infected symptomatic dogs/20 animals	(P-MAPA)/2.0 mg/Kg by IM at 3-day intervals during 45 days/10 animals	–	↑ CD8^+^ T cells, IL-2 and IFN-γ↓ IL-10	Santiago et al., 2013
Brazil	Naturally infected symptomatic dogs/30 animals	Recombinant cysteine proteinase of *L. infantum* (rLdccys1) + *P. acnes*/3 SC doses at 30-day intervals/10 animals	–	↑ IFN-γ; ↑ DTH; ↓ IL-10; ↓ spleen parasite load	Ferreira et al., 2014
Iran	*L. infantum* experimentally infected infection with 3 × 10^7^ amastigotes. Treatment starts at 90 dpi/12 animals	IMOD + amastigotes/2 mg/kg over 1 h at 2-day intervals during 30 days/4 animals	–	↓ IFN-γ, IL- 2, IL- 4 e IL-10. All animals remained positive in parasitological evaluation in spleen biopsy	Malmasi et al., 2014
Brazil	Naturally infected symptomatic dogs/16 animals	*Leishmania braziliensis* antigens + MPL (LBMPL vaccine)/3 series of 10 SC doses at 10-day intervals/10 animals	–	↑ CD3^+^ T lymphocytes and their subpopulations; ↑ NK cells and CD14^+^; ↓ CD21^+^ B lymphocytes; ↓ number and intensity of disease symptoms	Roatt et al., 2017
United States	Naturally infected asymptomatic dogs/495 animals	Leish-Tec® (*Leishmania* A2 protein + saponin)/3 SC doses at 14-day intervals/250 animals	–	↓clinical progression with ↓ mortality	Toepp et al., 2018
Brazil	Naturally infected symptomatic dogs/14 animals	LaSap (*Leishmania amazonenses* antigens + saponin)/5 SC doses at 7- day intervals/8 animals	–	Improvement in clinical status; ↓ IgG; ↑ lymphoproliferative capacity	Viana et al., 2018

*The arrows (↑ and ↓) indicate the increase and decrease in biomarker levels, respectively, when compared to control groups. dpi, days post infection; IV, intravenous route; SC, subcutaneous route; IM, intramuscular route; OR, oral route; ID, intradermal route*.

Immunomodulators have been described as triggering the immune system against *Leishmania* infection resulting in parasite control (Taslimi et al., 2016). Domperidone, for example, was able to induce clinical improvement in CVL in 86% of the animals with multiple clinical signs, with serum antibody titres decreased by 38% (Gómez-Ochoa et al., 2009). Moreover, the protein aggregate of magnesium–ammonium phospholinoleate–palmitoleate anhydride (P-MAPA) was used as a immunomodulator approach against CVL, inducing partial immunocompetence in symptomatic dogs (Santiago et al., 2013). Contrarily, the IMOD (Novel Herbal Immunomodulator Drug) used as immunotherapeutic treatment in experimental CVL did not trigger a proinflammatory immune response or induce parasite control, resulting in low therapeutic efficacy (Malmasi et al., 2014).

Vaccine therapy terminology has been employed in immunotherapy treatment, since the authors described the vaccinal antigens used for inducing cell-mediated immune response against CVL. Borja-Cabrera et al. (2004) evaluated the immunotherapeutic efficacy of FML-vaccine in asymptomatic dogs, which induced a positive DTH response in 79–95% of the animals and parasite control in bone marrow. Contrarily, vaccination with Leish111f (MML polyprotein) plus MPL®-SE failed to deter disease progression (Gradoni et al., 2005). Santos et al. (2007) administered enriched-Leishmune® vaccine (FML-Saponin) in symptomatic dogs, resulting in a reduction in clinical signs and parasitic burden on the liver, spleen, bone marrow, and blood. Immunotherapy using Leish-110f® with the adjuvant MPL-SE (Monophosphoril Lipid A), alone or in combination with Glucantime® (immunochemotherapy) in symptomatic dogs, was able to reduce the number of deaths, increase survival probability, and trigger specific cellular reactivity for parasite antigens (Miret et al., 2008). Beyond that, the recombinant polyprotein using Leish-111f® antigen with MPL-SE® provided a 75% cure rate, which was higher as compared to dogs treated with chemotherapy (64%) or immunochemotherapy (50%) (Trigo et al., 2010).

The immunotherapeutic protocol using *L. infantum* recombinant cysteine proteinase (rLdccys1) in combination with adjuvant *Propionibacterium acnes* induced high IFN-γ with low IL-10 cytokine production along with a reduction in the spleen parasite load (Ferreira et al., 2014). Notably, the vaccine composed of *L. braziliensis* antigens associated with MPL adjuvant (LBMPL vaccine) in symptomatic dogs was able to trigger increased CD3^+^ T lymphocytes and their subpopulations, a reduction in CD21^+^ B lymphocytes, and an increase in NK cells and CD14^+^ monocytes. Moreover, the dogs exhibited an important decline in the number and intensity of disease symptoms, increased body weight, reduced splenomegaly, and a drop in the parasite burden (Roatt et al., 2017). Similarly, Viana et al. (2018) demonstrated that *L. amazonensis* antigens, alone or in association with saponin (LaSap therapeutic vaccine), used in symptomatic dogs improved their clinical status, reduced IgG serum levels, and triggered a lymphoproliferative profile using *L. infantum* antigens, resulting in an outstanding reduction in parasite load. Furthermore, the vaccine Leish-Tec® (*Leishmania* A2 protein plus saponin adjuvant—Ceva Saúde Animal Ltda) used as immunotherapy in asymptomatic dogs induced a curtailment in clinical progression and in mortality (Toepp et al., 2018).

**Figure 2:**
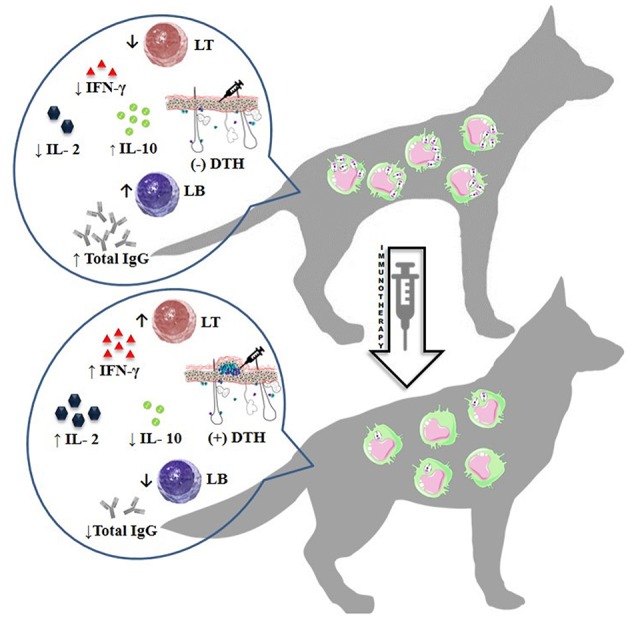
Immunotherapy-related immunological aspects. ↑, decreased; ↑, increased; IFN-γ, *Interferon gamma;* IL-2, Interleukin 2; IL-10, Interleukin 10; (+), positive; (–), negative; DTH, delayed-type hypersensitivity; IgG, Immunoglobulin G; LT, T lymphocyte; LB, B lymphocyte.

The different protocols used for immunotherapy or immunochemotherapy generally lead to an improvement in clinical signs with a possibility to further reduce the parasite burden by being activated in the immune system against *Leishmania* infection. Taken together, these results showed that immunotherapy is a promising strategy for the treatment of CVL. However, parasite clearance in CVL has not yet been achieved, irrespective of treatment, and this is the strongest negative aspect in these studies. The search for new immunotherapeutic agents to improve the results in this type of treatment is of great interest, given its aim to improve parasite control and develop approaches to blocking CVL transmission. All immunotherapy-related immunological aspects described above are summarized in Figure 2.

## Discussion and Perspectives: Immunotherapeutic Strategies to Treat and Block Canine Visceral Leishmaniasis Transmission

Although the immunotherapeutic protocols described were able to induce clinical improvement, there is still a major impasse when it comes to obtaining parasitological cure, as the *L. infantum*-infected dogs continue to be parasite reservoirs for sandfly vectors. Therefore, new protocols are needed to achieve a better efficacy in CVL treatment. Furthermore, innovative strategies can be incorporated into immunotherapy to interfere with the dynamics of disease transmission.

Considering that the sandfly's blood meal and the parasite's interaction with the invertebrate host are determining factors for *Leishmania* transmission, our research group has been developing studies focused on these factors so as to interfere with the parasite transmission dynamic (Graciano et al., 2019). The incorporation of vector antigens into new immunobiologicals is a promising strategy designed to disrupt the sandflies' life cycle, as well as block *L. infantum* transmission (Graciano et al., 2019). In fact, our research group has already identified different formulations with these capabilities that are currently being analyzed in addition to *Leishmania* antigens. The combination of parasite antigens with sandfly antigens in a single formulation as an immunotherapeutic protocol would provide more appropriate treatment. However, this new immunotherapeutic approach has not yet been tested in dogs. Finally, this type of immunotherapy could promote clinical improvement and efficient control of the parasite load, in addition to significantly reducing the risk of VL transmission and, thereby, lessening the number of canine and human cases.

## Author Contributions

AAG, JL, LR, and RM wrote the manuscript. AAG, PS, OM-J, HR, DO, DS, and TS reviewed the manuscript. AAG, AM, AG, OM-F, WD, DS-L, and RG drafted and critically evaluated the manuscript.

### Conflict of Interest

The authors declare that the research was conducted in the absence of any commercial or financial relationships that could be construed as a potential conflict of interest.
